# Hard flaccid syndrome: state of current knowledge

**DOI:** 10.1186/s12610-020-00105-5

**Published:** 2020-06-04

**Authors:** Maher Abdessater, Anthony Kanbar, William Akakpo, Sebastien Beley

**Affiliations:** 1 Department of urology, APHP -La Pitié-Salpêtrière University Hospital, 47-83 Boulevard de l’hopital, 75013 Paris, France; 2 Department of urology, Clinique Turin, Paris, France

**Keywords:** Male sexual dysfunction, Hard flaccid, Semi-rigid, Dysfonction sexuelle, syndrome de la détumescence rigide

## Abstract

**Introduction:**

Hard-flaccid syndrome is gaining increased interest among male sexual dysfunctions in the last years. It is poorly understood and defined. Most of the information comes from online forums. This paper is a review of current knowledge on the clinical presentation, diagnosis, pathophysiological mechanisms and treatments of this newly recognized condition.

**Material and methods:**

A literature review was conducted on MEDLINE, CENTRAL, PASCAL databases and google scholar, using the terms: hard, flaccid, syndrome. The research identified 16 articles published between 2018 and February 2019. After reference lists review and duplicates removal, 7 full text references were eligible and useful for our review that follows PRISMA guidelines.

**Results:**

The condition is acquired, chronic and painful. It is characterized by a constantly semi-rigid penis at the flaccid state and a loss in erectile rigidity. Patients have penile sensory changes, urinary symptoms, erectile dysfunction, pelvic floor muscles contraction and psychological distress. Symptoms are worse in standing position. The majority of the cases aged between their second and third decades. A traumatic injury at the base of an erect penis is the initial event. Neurovascular structures damage and subsequent sensory, muscular and vascular changes follow. Initial symptoms trigger emotional distress and reactional sympathetic stimulation that worsen symptoms. Diagnosis is based on patient’s history. Imaging and blood tests are normal. Differential diagnosis includes high-flow priapism and non-erecting erections. A multimodal treatment has been so far the most beneficial strategy, consisting of behavioral modifications to reduce stress and decrease pelvic floor muscles contraction, evaluation and treatment of the associated psychological conditions, and medical therapy for pain control and the treatment of the associated erectile dysfunction.

**Conclusion:**

Hard-flaccid syndrome is poorly recognized in the daily clinical experience and not well defined. A multimodal approach seems so far the most efficient strategy for treatment. Additional evidence based studies with better quality are needed to define the exact pathophysiological mechanisms and subsequently more efficient therapeutic strategies.

## Résumé

**Contexte:**

Le syndrome de la détumescence rigide (STR) fait le sujet de plusieurs discussions en ligne concernant les dysfonctions sexuelles. Cette entité est. mal définie et peu reconnue. Nous présentons dans cet article une revue de la littérature sur la présentation clinique, le diagnostic, les mécanismes physiopathologiques et les stratégies de thérapeutique du STR.

**Matériel et Méthodes:**

La revue de la littérature a été réalisée selon les recommandations PRISMA. En cherchant sur MEDLINE, CENTRAL, PASCAL et google scholar les termes « hard, flaccid et syndrome », on a identifié 16 articles publiés entre 2018 et février 2019. Après la revue des références des articles et le triage des doublons, 7 articles ont été retenus.

**Résultats:**

Il s’agit d’une pathologie acquise, chronique et douloureuse, caractérisée par une verge semi rigide à l’état flaccide avec une perte de rigidité lors des érections. Les patients souffrent de troubles sensitifs péniens et érectiles avec une contracture permanente des muscles pelviens ainsi que des troubles urinaires et psychologiques. Les symptômes sont aggravés par la position debout. La notion de traumatisme de la base de la verge est. rapportée par la majorité des patients. Ce traumatisme semble entrainer une altération des structures nerveuses et vasculaires péniennes à l’origine des troubles sensitifs et érectiles. Ces derniers créent des troubles émotionnels avec une stimulation sympathique réactionnelle qui engendre une contraction surajoutée des muscles pelviens et qui empire les symptômes. Le diagnostic est. clinique et les examens paracliniques sont normaux. Le traitement est. multimodal, il englobe les thérapies comportementales, la prise en charge des altérations psychologiques et de la douleur, afin d’agir sur la contraction des muscles pelviens et sur le stress associé à la dysfonction érectile.

**Conclusion:**

Le syndrome de la détumescence rigide reste peu connu et mal défini dans la pratique clinique. Une approche thérapeutique multimodale parait la plus adéquate actuellement. Des études supplémentaires sont nécessaires afin de mieux cerner cette entité dans le but d’améliorer sa prise en charge.

## Introduction

Male sexual dysfunctions (MSD) affect man’s sexual and social life and the wellbeing of couples [1]. They are not limited to erectile dysfunction (ED) and premature ejaculations. A new condition called hard-flaccid syndrome (HFS) is gaining increased interest in the last years and being the subject of many online forums. It is a chronic painful condition characterized by a semi-rigid penis at the flaccid state and a loss in erectile rigidity. Patients have penile sensory changes, erectile dysfunction, pelvic floor muscles contraction and psychological distress [1, 2]. HFS remains poorly understood, with no evidence based definition [3].

This paper is a review of current knowledge on the clinical presentation, diagnosis, pathophysiological mechanisms and treatments of this newly recognized condition.

## Material and methods

A literature review was conducted on MEDLINE, CENTRAL, PASCAL databases and google scholar, using the terms: hard, flaccid, syndrome. The review followed PRISMA guidelines. Neither language, date nor modality restrictions were applied.

The research identified 16 articles published between 2018 and February 2019. Reference lists of relevant articles were also reviewed for additional 1 article. After duplicates removal, 12 titles and abstracts were evaluated, from which 5 were excluded for having irrelevant subjects. Seven full text references were eligible and useful for our review, consisting of: 1 qualitative study utilizing thematic analysis of online forums and blogs, 1 comment on the previous article, 2 case series and 3 online web page articles. In total 6 cases were reported in the 2 case series and 152 online discussions were included in the thematic analysis.

The privacy of users was respected by Gul et al., who included only public forum sites consisting of anonymous users, and excluded the private discussions [4].

Quality of information provided by the websites was evaluated using the Health on the Net code (HONcode), the Journal of the American Medical Association (JAMA) benchmark criteria, and the DISCERN score, and results are detailed in Table 1. The HONcode certification was assessed as present or absent for each website. The JAMA benchmark criteria assessing website authorship, attribution, disclosure, and currency was rated on a scale between 0 and 4 points. The DISCERN score, that is based on 16 questions evaluating publication quality and reliability, varied between 0 and 80 points [5].

**Table 1:** Evaluation of the quality of the websites included

Score	Criteria	Websites		
		Urology news	PEGym	Entropy
HON code	Seal	No	No	No
JAMA benchmarks	Authorship	1	1	1
	Attribution	1	1	1
	Currencies	1	0	1
	Disclosure	1	0	1
	Total	4	2	4
DISCERN instrument	Are the aims clear?	5	3	5
	Does it achieve its aims?	5	3	5
	Is it relevant?	5	5	5
	Is it clear what sources of information were used to compile the publication (other than the author or producer)?	5	5	5
	Is it clear when the information used or reported in the publication was produced?	5	5	5
	Is it balanced and unbiased?	5	3	3
	Does it provide details of additional sources of support and information?	1	5	1
	Does it refer to areas of uncertainty?	5	5	1
	Does it describe how each treatment works?	5	3	5
	Does it describe the benefits of each treatment?	5	3	5
	Does it describe the risks of each treatment?	1	1	1
	Does it describe what would happen if no treatment is used?	1	1	1
	Does it describe how the treatment choices affect overall quality of life?	5	1	3
	Is it clear that there may be more than one possible treatment choice?	5	1	1
	Does it provide support for shared decision-making?	1	1	1
	Based on the answers to all of the above questions, rate the overall quality of the publication as a source of information about treatment choices	4	1	2
	Total	63	46	49

*HON* Health on the Net; *JAMA* Journal of the American Medical Association

**Fig. 1:**
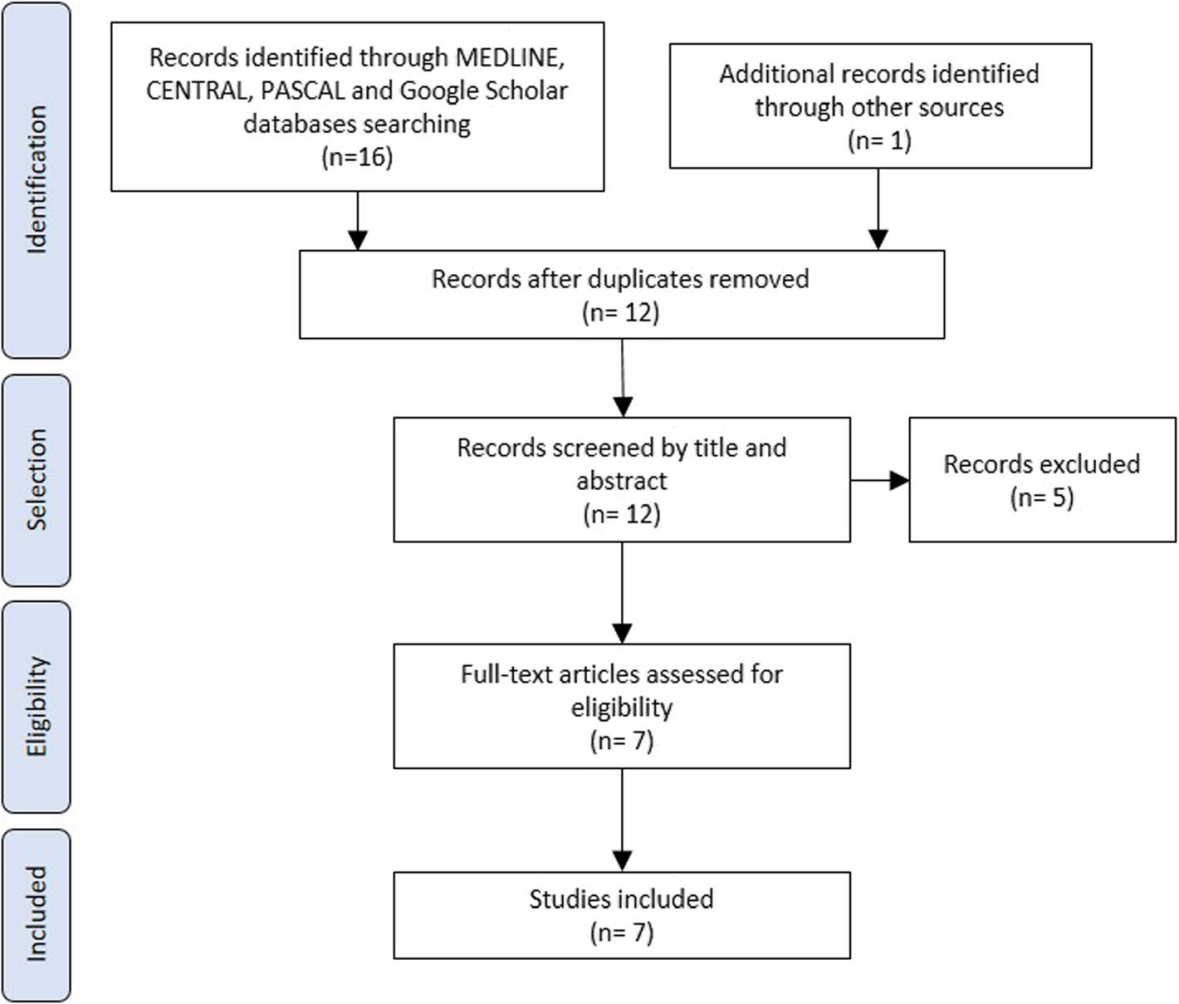
Flow diagram of the literature review process on hard flaccid syndrome

Results are presented in a descriptive manner. A flow diagram of the literature review process is presented in Fig. 1. Details and quality of the references included are presented in Table 2.

**Table 2:** Description of the details and the quality of the references included

Authors	Reference type	Quality assessment	Aspects discussed
Gul, 2019 [4]	Qualitative study utilizing thematic analysis of 152 online discussions from internet forums	Data limited to English literature Selection bias of internet users	Symptoms Initial event Management strategies Physiopathology diagram
Yachia, 2019 [6]	Comment on article		Differential diagnosis Relation to chronic pelvis pain syndrome
Gul, 2019 [1]	4 case series		Symptoms Pathophysiological mechanisms Treatment
Gul, 2019 [3]	2 case series		Symptoms
Hughes, 2019 [7]	Academic web page: Urology news	HONcode seal: negative JAMA benchmarks: 4 DISCERN score: 63	Clinical signs and symptoms Management
Bond, 2019 [8]	Commercial web page: Pegasus Gym Blog	HONcode seal: negative JAMA benchmarks: 2 DISCERN score: 46	Clinical presentation Physiopathology diagram
Harville, 2018 [2]	Health professional web page: Entropy Blog	HONcode seal: negative JAMA benchmarks: 4 DISCERN score: 49	Clinical symptoms Pathophysiological mechanisms Management

*HON* Health on the Net; *JAMA* Journal of the American Medical Association

## Results

### Clinical presentation

HFS is an acquired condition characterized by a constantly semi rigid penis at flaccid state. Patients report most commonly odd sensory changes in the penis best described by numbness or coldness with a decreased sensitivity, particularly at the level of the glans [1, 4]. Associated chief complaint is a new onset ED with decreased frequency of morning and nocturnal erections. Excessive visual and physical stimulation are needed to achieve erection, which are difficult to maintain. Typically, a reduction in rigidity is noted on erection, with soft and cold glans. Penile and perineal pain during micturition and ejaculation are reported, and in majority of patients, are worse in the standing position [4]. Patients suffer from emotional distress manifesting by anxiety, depression, decreased libido and insomnia, as it is the case with all the MSDs [4, 7]. Although urinary symptoms are uncommon, a reduction in the urinary flow have been reported [4]. Cases reported in literature age from their late teens to the seventh decade, but the majority are in their second or third decades [7]. Incidence is not well known, since most of the information on HFS is obtained from patients discussing their symptoms on online forums or private chats groups [4].

### Pathophysiological mechanisms

Pathophysiological mechanisms are not well defined. Most patients report that their symptoms started after a traumatic event (use of vacuum, tough masturbation or sex, jelqing, excessive squatting), with a varying delay from minutes to weeks. Several patients reported that the traumatic event happened while they were under the effect of drugs like marijuana and bremelanotide [1, 3].

A traumatic injury at the base of an erect penis, of the neurovascular structures, that supply the muscles of the pelvic floor and the penis, was suggested to be the initiating event [6, 7]. The injury of the dorsal artery of the penis, the bulbourethral and the pudendal arteries, and the pudendal and dorsal nerve of the penis cause vascular and sensory changes that are responsible of the partial engorgement of the penis during erections and the odd penile sensations described by the patients [6].

Initial symptoms trigger emotional distress and reactional sympathetic stimulation. The latter is responsible of prolonged pelvic floor muscles spasm that applies additional extrinsic compression of the neurovascular structures with consequent penile hypoxia, neuropraxia, additional sensory changes and impairment of the pelvic muscles [1, 7].

The altered motor function and sustained contraction of the ischiocavernous, bulbomembranous and the external urethral sphincter muscles contribute to the venous outflow obstruction of the penis that is responsible of the semi-hardness state of the flaccid penis. A secondary myoneuropathy with a loss of relaxation ability follows and consequent erectile and ejaculatory dysfunctions develop [1, 7].

**Fig. 2:**
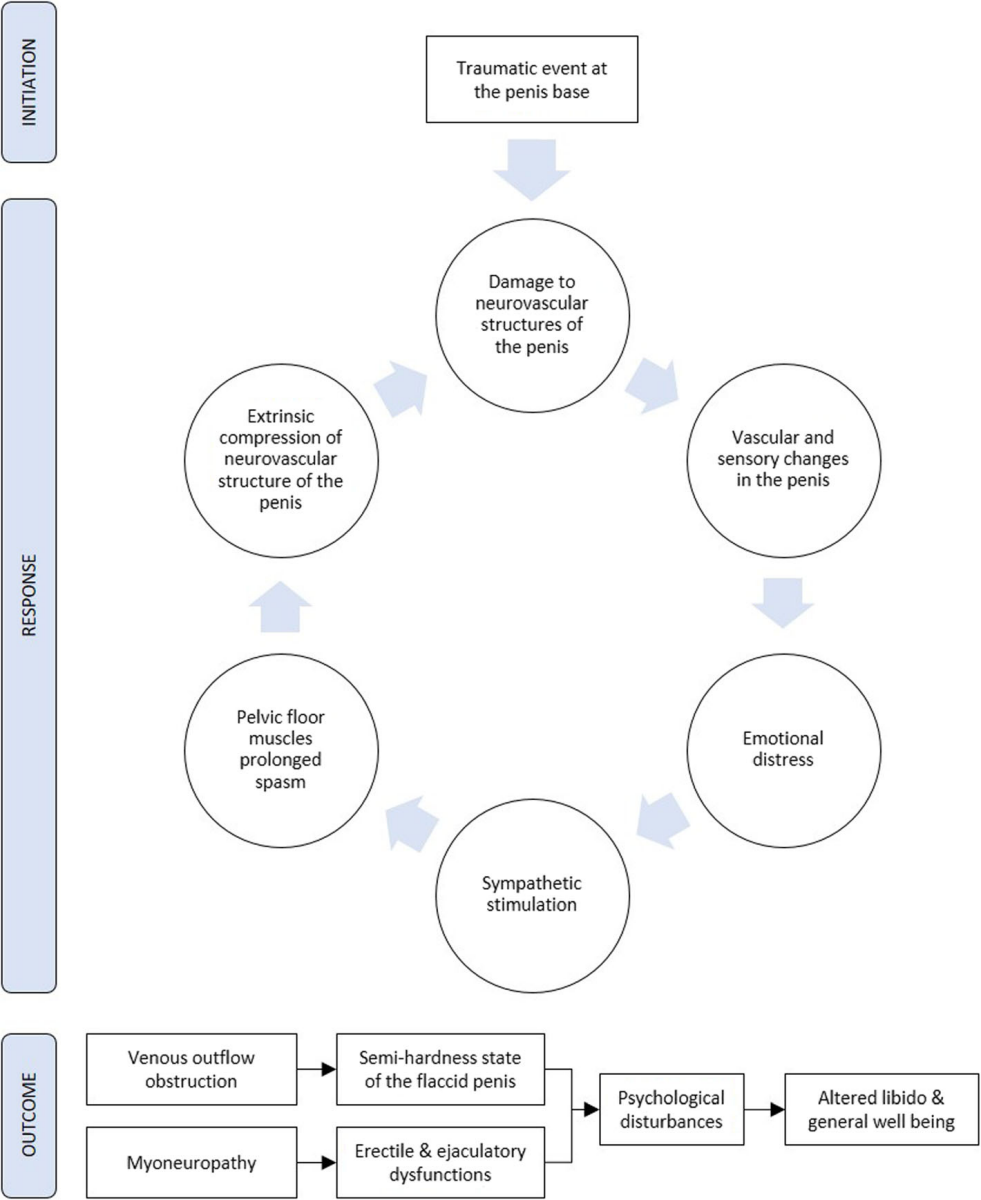
Diagram showing the pathophysiological mechanisms behind the hard flaccid syndrome

Symptoms result in psychological disturbances that affect the libido, erectile function, and general well-being [1]. Figure 2 describes suggests a pathophysiological scenario potentially involving most of the proposed mechanisms in literature.

The variety of the symptoms and their intensity are related to the location of the lesion and the extension of the inflammation of the injured nerves and vessels in the radix of the penis [1, 4].

### Diagnosis

The diagnosis is made based on patient’s history. Physical exam is unremarkable. Engorgement of the penis is revealed on examination. On erection, the glans might remain flaccid [7]. Hardness at the level of penis base is described by some [1, 4]. Imaging, doppler studies and blood tests, including hormonal studies, are normal [4].

### Differential diagnoses

Differential diagnoses include high-flow priapism (HFP) and non-erection erections. HFP is caused by arteriovenous fistulas in the penile vasculature, and is seen after blunt or penetrating trauma to the penis. Patients experience partial painless erections like those seen in HFS, but in HFP the doppler ultrasound can localize the rupture and the arteriovenous fistula [1]. Non-erecting erections were described by Yachia in 2009. They are caused by the deficiency or the absence of the suspensory ligament of the penis. Clinically this condition manifests by the lack of elevation of penis on erection. It is usually congenital or less frequently acquired after a trauma, and it is repaired surgically by corporopexy [6].

### Treatment

The treatment of this conditions is not well defined yet. Multiple therapeutic modalities were suggested, but were not equally efficient in all patients [4]. A multimodal treatment have so far been the most beneficial strategy [7].

The evaluation and treatment of the associated psychological conditions is crucial because stress and anxiety trigger additional sympathetic stimulation, and symptoms deterioration have been reported in periods of elevated stress [7]. Behavioral modifications (good sleep, healthy eating, regular exercises), biofeedback, cognitive behavioral therapies, breathing exercises, yoga reduce stress, improve well-being and decrease pelvic floor muscles contraction [7]. Pain can be controlled by analgesics. Medications like phosphodiesterase 5 inhibitors and antidepressants help treating associated erectile dysfunction and psychological conditions. Low-intensity shock wave therapy [1] has been temporarily alleviating in some patients who failed other therapies.

### Limitations

Our review was limited only to English and French references, and most of the analysis was obtained from internet forums. Although the collection of data from online patient’s forum is considered valid when literature is not available [9] and very useful for the description of the clinical aspects of a phenomenon [4], it has several limitations. First of all, selection bias is inevitable since only internet users are included. Another major drawback is deindividuation, because users tend to have more extreme and offensive tone on online blogs than they would in real life [9]. The online forum analysis lacks socio-demographic information which are of high importance when reviewing a medical pathology. Furthermore, the informed consent was not obtained from users who may be annoyed by the analysis of their posts. Overall, the quality of the websites included was not equal and not exclusively high. It was assessed using the HON code, the JAMA benchmark criteria, and the DISCERN score. None had a HON code certification, and the JAMA benchmarks and the DISCERN score varied between the three websites as detailed in Table 1.

## Conclusion

HFS has been the subject of discussion and debate on many male forums [8]. It is not recognized by sexual medicine community [1], and poorly recognized in the daily clinical experience [4]. Many patients are suffering from this conditions, and the majority is misdiagnosed and left untreated [7]. An initial traumatic event with the resulting inflammatory response is thought to generate stress that is a key factor is developing a prolonged contraction of the pelvic muscles. It is difficult to treat. A multimodal approach seems so far the most efficient strategy [8]. Physical therapy seems to play important role in the relaxation of the pelvic floor muscles [2]. Additional evidence based studies are needed to define the exact pathophysiological mechanisms and subsequently give more efficient therapeutic strategies.

## Data Availability

Not applicable.

## Abbreviations

ED: Erectile dysfunction
HFP: High-flow priapism
HFS: Hard flaccid syndrome
HON: Health on the Net
JAMA: Journal of the American Medical Association
MSD: Male sexual dysfunction

## References

[1] Gul M, Towe M, Yafi FA, Serefoglu EC (2020). Hard flaccid syndrome: initial report of four cases. Int J Impot Res.

[2] Harville M. Hard Flaccid Syndrome: Penetrating What We Know [Internet]. Entropy Physiother. 2018 [cited 2020 Feb 23]. Available from: http://entropy-physio.com/blog/hard-flaccid-syndrome-penetrating-what-we-know.

[3] Gül M, Serefoglu EC (2019). Hard flaccid: is it a new syndrome?. J Sex Med.

[4] Gul M, Huynh LM, El-Khatib FM, Yafi FA, Serefoglu EC. A qualitative analysis of internet forum discussions on hard flaccid syndrome. Int J Impot Res. 2019. 10.1038/s41443-019-0151-x. 10.1038/s41443-019-0151-x 31175339

[5] Fahy E, Hardikar R, Fox A, Mackay S (2014). Quality of patient health information on the internet: reviewing a complex and evolving landscape. Australas Med J.

[6] Yachia D. Comment on “A qualitative analysis of Internet forum discussions on hard flaccid syndrome.”. Int J Impot Res. 2019. 10.1038/s41443-019-0192-1. 10.1038/s41443-019-0192-1 31474756

[7] Hughes K, Parnham A, Lucky M (2018). Hard flaccid syndrome. Urol News.

[8] Bond J. Hard Flaccid: Beyond The Edge Of Science? [Internet]. 2019 [cited 2020 Feb 23]. Available from: https://www.pegym.com/articles/hard-flaccid-beyond-the-edge-of-science.

[9] Holtz P, Kronberger N, Wagner W (2012). Analyzing internet forums: a practical guide. J Media Psycho.

